# Fatal Transmissible Amyloid Encephalopathy: A New Type of Prion Disease Associated with Lack of Prion Protein Membrane Anchoring

**DOI:** 10.1371/journal.ppat.1000800

**Published:** 2010-03-05

**Authors:** Bruce Chesebro, Brent Race, Kimberly Meade-White, Rachel LaCasse, Richard Race, Mikael Klingeborn, James Striebel, David Dorward, Gillian McGovern, Martin Jeffrey

**Affiliations:** 1 Laboratory of Persistent Viral Diseases, Rocky Mountain Laboratories, National Institute of Allergy and Infectious Diseases, Hamilton, Montana, United States of America; 2 Electron Microscopy Section, Research Technology Branch, Rocky Mountain Laboratories, National Institute of Allergy and Infectious Diseases, Hamilton, Montana, United States of America; 3 VLA (Lasswade), Penicuik, Scotland, United Kingdom; University of Alberta, Canada

## Abstract

Prion diseases are fatal neurodegenerative diseases of humans and animals characterized by gray matter spongiosis and accumulation of aggregated, misfolded, protease-resistant prion protein (PrPres). PrPres can be deposited in brain in an amyloid-form and/or non-amyloid form, and is derived from host-encoded protease-sensitive PrP (PrPsen), a protein normally anchored to the plasma membrane by glycosylphosphatidylinositol (GPI). Previously, using heterozygous transgenic mice expressing only anchorless PrP, we found that PrP anchoring to the cell membrane was required for typical clinical scrapie. However, in the present experiments, using homozygous transgenic mice expressing two-fold more anchorless PrP, scrapie infection induced a new fatal disease with unique clinical signs and altered neuropathology, compared to non-transgenic mice expressing only anchored PrP. Brain tissue of transgenic mice had high amounts of infectivity, and histopathology showed dense amyloid PrPres plaque deposits without gray matter spongiosis. In contrast, infected non-transgenic mice had diffuse non-amyloid PrPres deposits with significant gray matter spongiosis. Brain graft studies suggested that anchored PrPsen expression was required for gray matter spongiosis during prion infection. Furthermore, electron and light microscopic studies in infected transgenic mice demonstrated several pathogenic processes not seen in typical prion disease, including cerebral amyloid angiopathy and ultrastructural alterations in perivascular neuropil. These findings were similar to certain human familial prion diseases as well as to non-prion human neurodegenerative diseases, such as Alzheimer's disease.

## Author Summary

Prion diseases, also known as transmissible spongiform encephalopathies, are infectious fatal neurodegenerative diseases of humans and animals. A major feature of prion diseases is the refolding and aggregation of a normal host protein, prion protein (PrP), into a disease-associated form which may contribute to brain damage. In uninfected individuals, normal PrP is anchored to the outer cell membrane by a sugar-phosphate-lipid linker molecule. In the present report we show that prion infection of mice expressing PrP lacking the anchor can result in a new type of fatal neurodegenerative disease. This disease displays mechanisms of damage to brain cells and brain blood vessels found in Alzheimer's disease and in familial amyloid brain diseases. In contrast, the typical sponge-like brain damage seen in prion diseases was not observed. These results suggest that presence or absence of PrP membrane anchoring can influence the type of neurodegeneration seen after prion infection.

## Introduction

Transmissible spongiform encephalopathies (TSE diseases) or prion diseases are fatal neurodegenerative diseases of humans and animals. These diseases include scrapie in sheep, bovine spongiform encephalopathy (BSE) in cattle, and chronic wasting disease (CWD) in cervids, as well as several human diseases including kuru, Gerstmann-Sträussler-Scheinker syndrome(GSS), and sporadic, familial and variant forms of Creutzfeldt-Jakob disease (CJD) (see [1] for review). TSE diseases are transmissible within a species, but can also cross to new species in some cases. For example, variant CJD appears to be a form of BSE transmitted to humans. In addition, experimental transmission to rodents such as mice, hamsters and, more recently, bank voles [2],[3], has provided numerous models for laboratory research.

In prion diseases brain pathology is characterized by spongiform degeneration of the gray matter together with neuronal loss and gliosis. During disease there is an accumulation in brain of an abnormal partially protease-resistant form of prion protein (PrPres) derived from host-encoded protease-sensitive prion protein (PrPsen). PrPres can be detected by immunoblot or immunohistochemistry, and this detection is often used as an important diagnostic feature of prion disease. PrPres can be deposited in brain either as large fibrillar amyloid plaques and/or as small diffuse punctate deposits of non-amyloid aggregated protein. The diffuse non-amyloid PrPres form is prevalent in many human sCJD cases and most prion disease animal models [4]–[7]. However, both amyloid and non-amyloid forms of PrPres coexist in some human and animal prion diseases [6] [8]–[13], and both forms may contribute to prion disease pathogenesis.

A variety of proteins are capable of forming amyloid deposits in nervous system tissues as well as other organs. Amyloid deposits often displace organ structure resulting in dysfunction and cell death. In cerebral amyloid angiopathy (CAA), associated with Alzheimer's disease (AD) and several genetic CNS amyloid diseases, vascular amyloid deposits can damage the structure of blood vessel walls leading to hemorrhage or thrombosis [14],[15]. However, in AD, oligomeric pre-amyloid Aß aggregates are also thought to have important neuropathogenic effects. For therapy of diseases such as AD and prion diseases, where both amyloid and non-amyloid may be pathogenic, it will be important to understand the contribution of both types of abnormal protein aggregates to the various pathogenic processes present in these complex diseases. Therefore, we focused on the pathogenic effects of PrPres amyloid versus non-amyloid induced by prion infection in mice.

In uninfected animals PrPsen is anchored to the plasma membrane by a glycosylphosphatidylinositol (GPI) moiety [16]. In prion disease PrPres is found on plasma membranes of neurons and other brain cells, where it is associated with morphological membrane changes that are common to different animal TSEs [7] [17],[18]. Membrane attachment of PrP may have an important influence on the prion disease process. To study the role of PrP membrane linkage on pathogenesis of prion disease we previously generated 2 lines of transgenic mice (tg44+/− and tg23+/−), which express PrP lacking the GPI anchor at similar levels and do not express GPI membrane–anchored PrP. Anchorless PrP in these mice is secreted by cells and is not attached to the plasma membrane [19]. After scrapie infection, both lines of transgenic mice developed high titers of prion infectivity and extensive PrPres amyloid deposits in brain at late times after infection; however, typical scrapie clinical signs and gray matter spongiosis characteristic of prion diseases were not seen [19].

In the present paper we studied homozygous anchorless PrP transgenic mice, which expressed two-fold more anchorless PrPsen than the above mentioned heterozygous transgenic mice. In these experiments scrapie infection of homozygous mice produced a fatal clinical disease. However, this disease differed in incubation period, clinical signs, and neuropathology from typical prion disease seen in non-transgenic mice, which express anchored PrP, and thus appeared to be a distinct pathogenic process. Therefore, depending on the presence or absence of anchored PrP, scrapie infection could induce two different fatal brain diseases: PrPres amyloidosis without gray matter spongiosis in anchorless PrP transgenic mice, and diffuse non-amyloid PrPres with gray matter spongiosis in mice with anchored PrP.

## Results

### Brain PrPsen levels in anchorless PrP transgenic mice and non-transgenic mice

Since PrPsen expression is known to influence scrapie incubation period [20] [21], it is possible that low PrP expression might account in part for the lack of clinical scrapie disease in previous experiments using heterozygous tg44+/− and tg23+/− mice [19]. Therefore, in the present study we generated homozygous anchorless PrP transgenic mice from both lines 44 and 23. These mice each expressed two anchorless PrP transgene alleles and no normal mouse PrP alleles.

**Figure 1:**
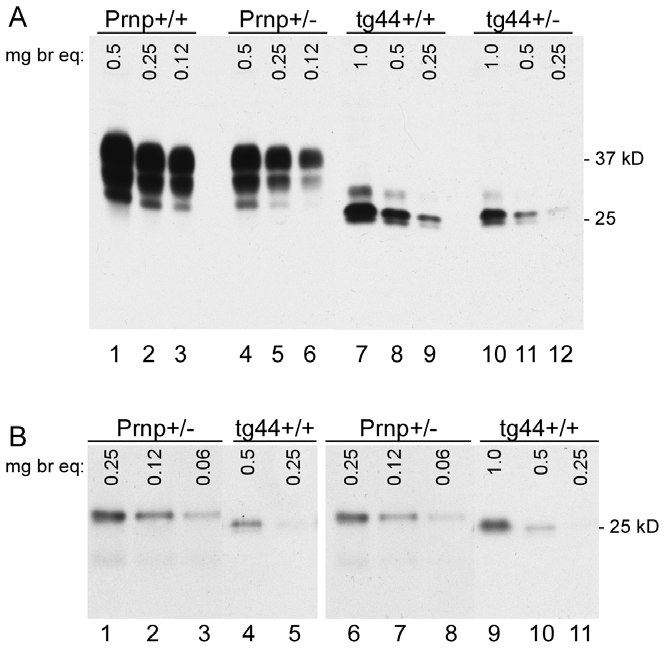
Brain PrPsen expression levels. C57BL/10 (Prnp+/+), Prnp+/−, expressing normal anchored mouse PrP, and transgenic mice (tg44+/+ and tg44+/−), expressing only anchorless mouse PrP were compared. Immunoblots were done using brain homogenates made as described in methods, and samples were serially diluted two-fold in sample buffer to give the mg brain equivalents shown on the figure. Bands were detected with monoclonal antibody D13. (A) No PNGase digestion. Lanes 1–3: Prnp+/+, Lanes 4–6: Prnp+/−, Lanes 7–9: tg44+/+ and Lanes 10–12: tg44+/−. (B) After PNGase F treatment. Lanes 1–3: Prnp+/−, Lanes 4–5: tg44+/+, Lanes 6–8: Prnp+/− and 9–11: tg44+/+ mice. Lanes 1–5 were from one experiment and 6–11 were from a separate experiment and results shown are for 4 different mice. Lower apparent molecular weight in PrP of transgenic mice is due to lack of the GPI anchor. Prnp+/− mice appeared to have 4-fold more brain PrPsen than tg44+/+ mice (compare lanes 2 and 4, also lanes 7 and 10). Data shown are for tg44+/+ and tg44+/− mice. By immunoblot PrPsen expression levels in tg23 mice were indistinguishable from those in tg44 mice (data not shown).

PrPsen levels were analyzed by immunoblotting in brain homogenates of uninfected transgenic and non-transgenic mice. Homozygous tg44+/+ mice expressed 2-fold higher levels compared to tg44+/− mice (Figure 1A). Non-transgenic C57BL/10SnJ mice homozygous for the PrP gene (Prnp+/+) were used as controls, and these mice expressed 2-fold higher PrP levels than did Prnp+/− mice (generated by crossing Prnp+/+ mice to Prnp-null mice also on the C57BL/10SnJ background (see methods)) (Figure 1A). Quantitative comparisons between transgenic and non-transgenic mice were difficult due to PrP glycosylation differences (Figure 1A). Therefore we compared these mice using PrPsen deglycosylated with PNGase F (Figure 1B). In these experiments Prnp+/− mice had approximately four-fold higher PrPsen levels than tg44+/+ mice (compare lanes 2 vs. 4 and lanes 7 vs. 10). Given the 2-fold difference between Prnp+/+ and +/− mice, tg44+/+ expressed 8-fold lower levels of brain PrPsen than did Prnp+/+ mice. Brain PrPsen levels in tg44+/+ and tg23+/+ mice were indistinguishable (data not shown).

### Scrapie-induced clinical disease in transgenic and non-transgenic mice

**Figure 2:**
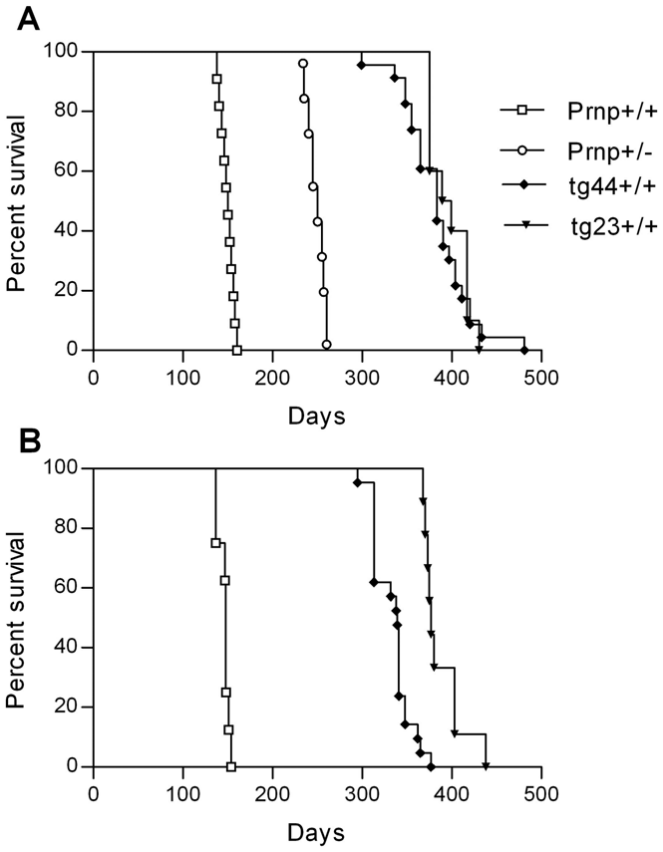
Survival curves for scrapie-infected mice. Transgenic mice (tg44+/+ and tg23+/+) expressing only anchorless PrP, C57BL/10 (Prnp+/+) and Prnp+/− mice, expressing normal anchored mouse PrP were compared. Mice were inoculated intracerebrally with 22L scrapie (panel A) and RML scrapie (panel B) and observed weekly for development of disease (see Table 1). Mice were euthanized when clinical signs were severe as described in Table 1. N values for each group are as follows: Panel A (22L): Prnp+/+, 11; Prnp+/−, 17; tg44+/+, 23; tg23+/+, 10. Panel B (RML): Prnp+/+, 8; tg44+/+, 21; tg23+/+, 9.

Scrapie-induced clinical disease was analyzed in transgenic and non-transgenic mice using two different scrapie strains, RML and 22L. After intracerebral (IC) inoculation of scrapie strain 22L, Prnp+/+ and +/− mice developed clinical scrapie at 150–165 dpi and 245–260 dpi respectively (Figure 2A). Similar results were also seen using the RML scrapie strain in Prnp+/+ mice (Figure 2B). Prnp+/− mice were not tested with the RML strain. In contrast to experiments with non-transgenic mice, all tg44+/+ and tg23+/+ mice infected with strains 22L or RML developed neurological signs and required euthanasia from 300 to 480 dpi (Figure 2). Homozygous transgenic mice differed from non-transgenic mice in incubation period, duration and progression of clinical signs, as well as gait and postural abnormalities (Table 1). The most obvious signs in homozygous transgenic mice were the presence of a wide-based gait, rear extremity weakness with low posture, and lack of kyphosis. The signs seen in homozygous transgenic mice differed from the narrow-based tippy-toed gait and frequent kyphosis seen in infected Prnp+/+ and +/− mice (Table 1). The gait and postural differences probably reflected different patterns of neurological damage. Overall the differences between non-transgenic mice and homozygous transgenic mice suggested that there might be different pathogenic mechanisms operating in these two scrapie-induced disease models.

**Table 1:** Clinical aspects of disease in 22L or RML scrapie-infected C57BL/10 and tg44+/+ mice.

Clinical Manifestations	C57BL/10	Tg44+/+^a^
Incubation period (days)	150–160	300–480
Duration of clinical signs (days)	15–25	30–60
Progression of signs	steady	erratic^b^
Kyphosis	most^c^	few^c^
Narrow-based tip-toe gait	most	few
Splayed hind legs, wobbly gait	few	most
Low body posture	few	most
Weight loss	all	all
Ungroomed coat	all	all
Hypoactive (late)	all	all
Rear leg clasping response	most	most

a Clinical signs were similar in infected tg44+/+ and tg23+/+ mice infected by IC inoculation with either 22L or RML scrapie strains as described in the methods. End-stage neurological disease in these mice was characterized by profound weakness, hypoactivity and reduced ability to eat or drink, requiring euthanasia. In heterozygous tg44+/− and tg23+/− mice clinical signs were similar, but less severe and more erratic. However, most heterozygous mice developed dermatitis, bladder distention, infection and tumors requiring euthanasia prior to development of end-stage neurological disease.

b Clinical signs were present on an erratic basis initially, but became more consistent and severe over time.

c Most = 85–100%; few = 0–15%.

In our earlier studies, heterozygous tg44+/− and tg23+/− mice did not manifest the usual clinical signs of scrapie during 600 days of observation after infection with scrapie strains 22L or RML (Table 1) [19]. However, in the present experiments with the experience of observing the clinical signs described above in homozygous transgenic mice, we noted similar clinical signs in the infected heterozygous transgenic mice starting around 480 dpi. Clinical diagnosis in these mice was difficult due the erratic presence of signs in the initial stages, the longer duration of signs, and the possibility of confusion with signs of old age. These mice were euthanized between 480 and 700 dpi, but the indication for euthanasia was primarily debilitation (weight loss, dermatitis, bladder distention, cancer and infections), rather than neuromuscular dysfunction.

### PrPres levels

**Figure 3:**
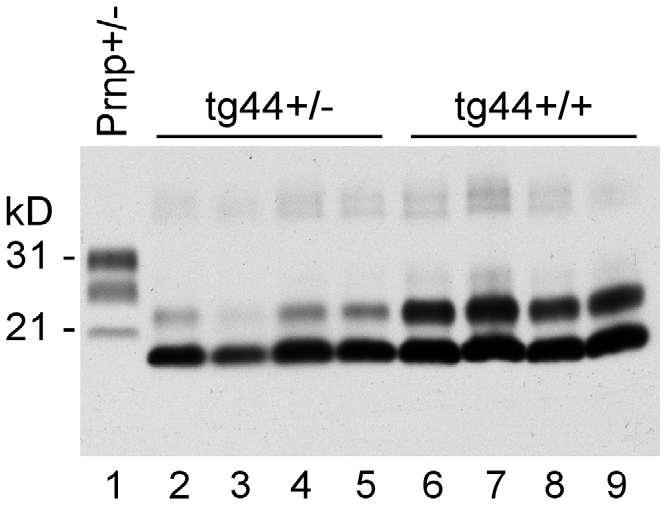
Detection of PrPres by immunoblot using monoclonal antibody D13. Comparison of PrPres in brain of 22L scrapie-infected tg44+/+ and tg44+/− mice at the time of clinical disease. All lanes were loaded with 0.25 mg brain tissue equivalents. A clinical Prnp+/− mouse is shown for comparison. Lane 1: Prnp+/− (251 dpi), PrPres bands are seen at 21, 28, and 31 kD; lanes 2–5: tg44+/− mice (567, 589, 594, 594 dpi) and lanes 6–9 tg44+/+ (348, 365, 384, 408 dpi). PrPres bands are at 18 and 23 kD. The sizes are lower in these mice due to lack most carbohydrates and lack of GPI [19]. PrPres levels in tg44+/− mice were approximately 50% lower than in tg44+/+ mice.

PrPres deposition in brain is a major hallmark of prion diseases, and PrPres is often associated with areas of brain degeneration. Therefore brain PrPres levels in scrapie-infected mice were analyzed by immunoblot. As shown in Figure 3, tg44+/+ mice with clinical neurological disease had higher amounts of PrPres at 348–408 dpi than tg44+/− mice had at 567–594 dpi, which was when these mice had to be euthanized due to debilitating signs as described in Table 1. A similar difference was seen between tg23+/+ and tg23+/− mice (data not shown). The difference between tg44+/+ and tg44+/− mice in timing and levels of PrPres correlated with the higher PrPsen expression level seen in homozygous mice (Figure 1A), and appeared to explain the earlier onset and more prominent clinical signs seen in homozygous transgenic mice.

The PrPres detected in these immunoblots had a molecular weight of 19 kD. In our earlier study this band was found to react with an anti-PrP peptide antibody (R20) directed at the C-terminal region of PrP (residues 218–232) [19]. Therefore, there was no evidence for loss of these C-terminal residues as often occurs in human GSS [6],[22].

Interestingly tg44+/+ and tg44+/− mice had higher PrPres levels than did non-transgenic Prnp+/+ or +/− mice, but the non-transgenic mice died earlier than the tg44+/+ mice (Figure 2). This suggested either that amyloid PrPres in tg44+/+ mice might be less pathogenic than the non-amyloid PrPres in non-transgenic mice, or that transgenic mice might be less susceptible to the pathogenic effects of PrPres amyloid due to the absence of membrane-anchored PrP.

### Infectivity levels in tg44 mice

Previously we showed that scrapie-infected tg44+/− mice lacked signs of clinical scrapie but had infectivity titers in brain as high as 4.6×10^8^ ID50/gram brain at 120–286 dpi as measured by end-point titration in C57BL/10 mice [19]. In the present experiments we passaged brain from infected tg44+/− (passage 1) mice into other tg44+/− mice (passage 2), and at 512 and 531 dpi we found brain infectivity titers of 1.1–1.3×10^10^ ID50/gram brain (Table 2). These mice also had brain PrPres levels similar to those shown in other tg44+/− mice (Figure 3). Similar high titers were detected in passage 1 homozygous tg44+/+ mice at 384 dpi (Table 2). Thus in the transgenic anchorless PrP model, scrapie infectivity was present in brain at very high titers. Furthermore, the agent did not appear to develop new strain-like properties selective for tg44+/− mice as it could passage easily from tg44+/− mice to either C57BL/10 or tg44+/− mice.

**Table 2:** Infectivity titers of 22L scrapie from brains of Tg44 mice after 1 or 2 passages.

Tg44 zygosity^a^	Passage^b^	Days^c^	Titer^d^
+/−	1	286	1.1×10^9^
+/−	2	512–531	1.1–6.3×10^10^
+/+	1	384	>1×10^9^ e

a Zygosity of tg44 transgene in scrapie-infected mouse whose brain infectivity was titered.

b Number of passages in tg44 mice (1 = first; 2 = second). For passage 1, tg44+/− and tg44+/+ mice were inoculated intracerebrally with a 22L scrapie stock derived from C57BL/10 mice with a titer of 1.4×10^9^ ID_50_per gram of brain tissue. For the second passage in tg44+/− the brain of the 286 dpi passage 1 tg44+/− mouse was used as the inoculum. The preliminary titration of the passage 1 mouse from 286 dpi was previously reported to be >2.4×10^8^ ID_50_per brain [19].

c Days post-inoculation when brain was harvested for titration.

d Brain titer was determined by end-point dilution titration in C57BL/10 mice for tg44+/− or in tga20 mice for tg44+/+. Titer was expressed as ID_50_per gram of brain tissue.

e The titration of this brain is still in progress.

### Histopathology and PrPres distribution detected by IHC

**Figure 4:**
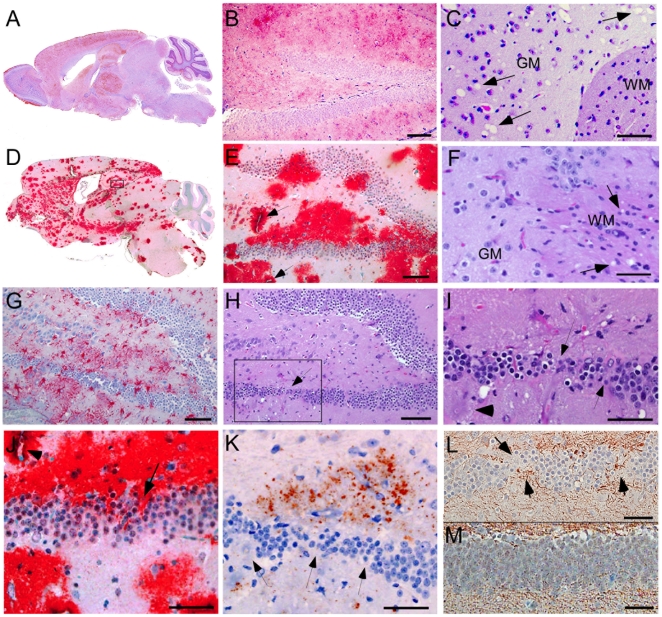
Light microscopic histopathology of scrapie-infected C57BL/10 and tg44+/+ transgenic mice. (A) Whole brain sagittal section of C57BL/10 mouse infected IV with RML scrapie at 180 dpi showing wide distribution of diffuse PrPres stained with monoclonal antibody D13. (B) High power view of C57BL/10 mouse infected IC with RML scrapie at 157 dpi showing diffuse punctuate pattern of PrPres in hippocampus. (C) H&E stain of forebrain of C57BL/10 mouse infected IP with 22L scrapie at 375 dpi. Anterior commissure white matter (WM) is seen in lower right. Numerous scrapie vacuoles (arrows) are visible in surrounding gray matter(GM). (D) Whole brain saggital section of tg44+/+ mouse D554 infected IC with RML scrapie showing dense plaque-like PrPres stained with monoclonal antibody D13 at 341 dpi. PrPres was found in most CNS areas including cerebral cortex, corpus callosum, forebrain, hippocampus, thalamus, hypothalamus, midbrain, colliculi, brainstem, and spinal cord. Cerebellar involvement was minimal after RML infection, as shown in panel 4D, but was strong in cerebellar molecular layer, granular layer and meninges after 22L infection (not shown). (E) Higher power of panel D shows large dense PrP plaques surrounding dentate gyrus of hippocampus often in a perivascular distribution (arrows). Note difference compared to diffuse PrPres staining in panel B. (F) H&E stain of mouse D554 showing vacuoles in the white matter (WM) near the anterior commissure and no vacuoles in surrounding gray matter, i.e. opposite distribution of vacuoles compared to C57BL/10 mouse in panel C. (G) Astrogliosis seen by staining with anti-GFAP in dentate gyrus of mouse D554. (H) H&E stain of dentate gyrus of mouse D554 shows marked neuronal loss in lower arm of gyrus (box and arrow). Boxed outlines region shown in panel I. (I) High power view of lower arm of dentate gyrus outlined in panel H, shows multiple areas of neuronal loss (arrows). Plaques surround this area and one plaque is indicated with the arrowhead at the left. (J) High power view of area shown in panel I shows D13 staining of PrPres plaques impinging on damaged neurons of the dentate gyrus (arrow). Arrowhead shows plaque around blood vessel in upper left corner. (K) Deposition of amyloid precursor protein, APP, (red-brown stain) adjacent to area of neuronal loss (arrows) in dentate gyrus of same area shown in panels I and J. (L) Abnormal axonal proliferation shown by staining with anti-neurofilament protein in area of neuronal loss (arrows) in dentate gyrus of mouse D554. (M) Anti- neurofilament protein staining of dentate gyrus of uninfected control mouse shows no neuronal damage or abnormal axonal staining within the gyrus. Scale bars in panels B, E, G, and H are 100 microns; all other scale bars are 50 microns. Similar pathological changes were seen at the time of clinical disease in both tg44+/+ and tg23+/+ mice infected with either 22L or RML strains of scrapie. D13 staining of PrPres in panels E and J was similar to results described previously in tg44+/− and tg23+/− mice at ≥498 dpi [19].

We compared the PrPres deposition and neuropathology after scrapie infection in transgenic tg44+/+ mice and non-transgenic C57BL/10 control mice (Table 3). Following scrapie infection in C57BL/10 mice typical TSE-specific diffuse deposits of PrPres were found in many brain areas (Figure 4A, 4B). This PrPres did not stain with the amyloid stain, Thioflavin S [19]. In many brain regions by H&E staining we observed gray matter spongiosis (Figure 4C), which is an important feature of TSE/prion diseases.

**Table 3:** Comparison of histopathological features in scrapie-infected C57BL/10 mice and homozygous anchorless PrP transgenic mice (tg44+/+).

Method	Lesion	TSE-specific	C57BL/10 mice	Tg44+/+ mice
Light microscopy	Protease-resistant PrP^a^	yes	fine^b^	coarse^b^
	PrP amyloid (Thioflavin S)^b^	yes	no	yes
	Distortion of brain by amyloid^c^	no	no	yes
	White matter vacuoles	no	rare	yes
	APP upregulation (a-APP)^d^	no	rare	yes
	Axonal dystrophy (a-NFP)^d^	no	rare	yes
	Neuronal loss	no	yes	yes
	Astrogliosis & microgliosis	no	yes	yes
	Gray matter vacuoles^e^	no	yes	rare
Electron microscopy	Membrane abnormalities^f^	yes	yes	no
	Dystrophic neurites and axons	no	rare	yes
	Neuritic and glial swelling	no	rare	yes
	Degenerate myelinated processes^g^	no	rare	yes

a Partially protease-resistant PrP (PrPres) is a hallmark of TSE/prion diseases. Most researchers including ourselves rely on the correlation between the immunoblot detection of PrPres and the immunohistochemical detection of PrP with staining properties and regional distribution found only in prion-infected animals, and thus refer to such disease-associated PrP as PrPres or PrPd.

b In most mouse scrapie models PrPres has a diffuse fine punctate staining pattern which is negative for staining with the amyloid stain, Thioflavin S. In both homozygous and heterozygous anchorless PrP transgenic mice the PrPres has a coarse dense plaque-like staining pattern and is Thioflavin S-positive.

c Distortion of neuroanatomical features such as the hippocampal and cerebrocortical neuronal layers was associated with the presences of large PrPres amyloid plaques. Such distortion was accompanied by neuronal loss (Figure 4H–L). This feature was seen in tg44+/+ and tg23+/+ mice, but was less frequent in heterozygous transgenic mice. Brain distortion appeared to be the result of the large amounts of PrPres amyloid present in homozygous transgenic mice (Figure 3).

d Abnormal staining with anti-APP indicates axonal or neuronal damage. This is easily seen in infected tg44+/+ mice, but is minimal in infected C57BL/10 mice. Antibodies reactive with phosphorylated or non-phosphorylated neurofilament protein (NFP) both showed abnormal staining in infected tg44+/+ mice compared to C57BL/10 mice (Figure 4L vs 4M).

e TSE vacuoles in gray matter are a hallmark of classical prion diseases. At the ultrastructural level they are characterized by fragmentation of the inner limiting membrane and presence of membrane fragments hanging into the vacuole, and by electron microscopy PrPres detectable by immunogold labeling is not found to be colocalized with these vacuoles ([13], [17], [7]).

f Several ultrastructural abnormalities, including spiral membranes, invaginated membranes, increased and fused clathrin-coated pits and membrane microfolds, have been found to be unique to TSE diseases, all of which co-localize with disease-associated membrane accumulations of PrPres. These changes are present in mice infected with different murine scrapie strains [17], in sheep scrapie [18] and in cattle BSE [7], and were not seen in infected homozygous or heterozygous tg44 or tg23 mice.

g In white matter distended empty myelin sheaths were seen, and these were often large enough to be noted as “vacuoles” by light microscopy.

**Figure 5:**
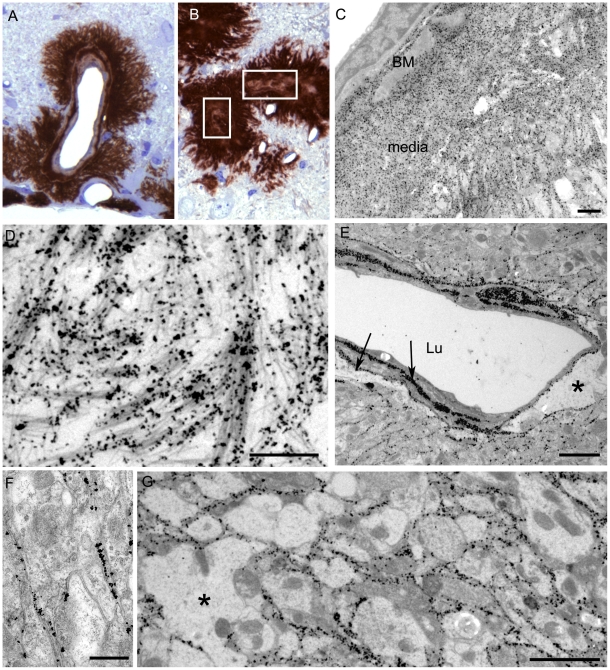
Immunological detection of PrPres in brain at both light and electron microscopic levels. The 22L scrapie-infected anchorless PrP tg44+/+ mouse shown was clinically positive at 377 dpi. (A–B) Light microscopy of 1 µm thick plastic-embedded tissue labelled with monoclonal antibody 1A8. (A) shows intravascular and perivascular PrPres. In (B), the marked vascular amyloid infiltration is associated with occlusion of the vascular lumen (boxes indicate occluded lumens of two vessels). When these vessels were visualised in the electron microscope the smooth muscle of the vascular media was totally replaced by amyloid and an amorphous electron dense material filled the lumen (not shown). (C–F) Electron microscopy. (C) Low power view of large PrPres amyloid plaque adjacent to a small artery. Vessel lumen is in upper left corner and an endothelial cell with a prominent nucleus is to the right of the lumen. Silver enhanced gold-labeled PrPres is seen within the basement membrane (BM) and within the heavy amyloid accumulation which partially replaces the smooth muscle media layer. Amyloid bundles radiate away from the vessel and through the neuropil at the bottom right. (D) A high magnification illustration of (C) showing PrPres labelling on small bundles of amyloid fibrils at the periphery of the plaque. (E) Marked PrPres accumulation at the endothelial and pericyte basement membranes (arrows) and extending into narrow extracellular spces between nearby neurites and perivascular glial processes. Asterix (*) shows area of astrocytic cytoplasmic swelling. Lu; lumen. (F) Neuropil of cerebrum showing immunogold label for PrPres present over the extracellular spaces between neurites bounded by pairs of adjacent plasmalemmae. No visible amyloid fibrils were visible and the spaces between cellular processes were regular and even. This non-fibrillar PrPres labelling which dissects between neurite and glial cell profiles may extend over large area of neuropil as shown in (G). Asterix (*) on left side shows enlarged glial process with loss of normal cytoplasmic organelles. Similar findings were observed in tg44+/+, tg44+/− and tg23+/− mice. Tg23+/+ mice were not examined by electron microscopy. Scale bars: A and B, 20 µm; C and D, 1 µm; E and G, 2 µm; F, 500 nm.

In scrapie-infected tg44+/+ mice PrPres accumulated as large dense plaque-like deposits, usually in a perivascular location around capillaries, veins and arteries in numerous brain regions, including leptomeninges, cerebral cortex, corpus callosum, forebrain, hippocampus, thalamus, hypothalamus, midbrain, colliculi, brainstem, and spinal cord (Figure 4D, 4E, 5A, 5B) After infection with the 22L scrapie strain, the cerebellar molecular layer and granular layer were also involved [19], but this was not seen after infection with the RML strain (Figure 4D). These deposits were Thioflavin S-positive [19], and no areas of diffuse non-amyloid PrPres were observed. The most distinguishing histopathological feature in tg44+/+ mice at the time of clinical signs was distortion of brain structures adjacent to large amyloid plaques in many areas (Figures 4D, E, H–L). These areas had intense micro- and astrogliosis (Figures 4G). Small blood vessels showed occasional micro-hemorrhages, or perivascular haemosiderin accumulation, but no lymphocyte infiltration of blood vessel walls was detected. Marked neuronal loss was seen around edges of some gray matter plaques (Figure 4H, I); however, no gray matter spongiosis typical of prion diseases was seen (Figure 4C vs 4F). In addition, tg44+/+ mice had focal areas of abnormal staining of amyloid precursor protein (APP) (Figure 4K), non-phosphorylated neurofilament protein (NFP) (Figure 4L), and phosphorylated NFP (not shown), all of which indicated a process of severe axonal dystrophy. These latter effects were rarely seen in scrapie-infected C57BL/10 mice (Table 3). These results suggested that scrapie-infected anchorless PrP transgenic mice had a different pathogenic process compared to non-transgenic C57BL/10 mice.

### Ultrastructural analysis of brain from infected tg44+/+ mice

For higher resolution of details, scrapie-infected tg44+/+ mice were also studied using immunohistochemistry on 1 micron thick plastic-embedded sections as well as immunogold labeling at the ultrastructural level. Light microscopy on thin sections showed both perivascular and vascular PrPres labeling (Figure 5A), as well as occlusion of vessels in some cases (Figure 5B). By electron microscopy abundant PrPres labeling of blood vessels was seen most predominantly at basement membranes. In some larger vessels smooth muscle cells of the media were atrophied and replaced by extensive PrPres accumulation (Figure 5C). Vascular and plaque PrPres accumulation could be seen to be of a fibrillar amyloid nature at high magnification (Figure 5D). In smaller vessels PrPres was seen at both endothelial and pericyte basement membranes (Figure 5E). PrPres was also observed within the extracellular space along the borders of swollen astroglial and neurite processes in the absence of visible fibrillar amyloid (Figure 5F and G). No PrPres labeling was seen in uninfected control mice.

**Figure 6:**
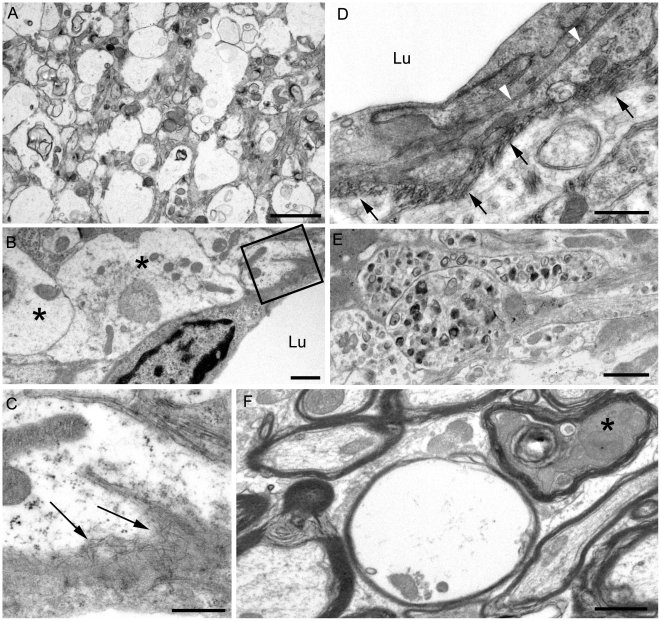
Ultrastructure of cerebral cortex and cerebellum of a 22L scrapie-infected tg44+/+ mouse. Sections were from same mouse as shown in Figure 5, and were stained with uranyl acetate/lead citrate. (A) Area of neuropil with severe vacuolation where most vacuoles originate within processes and are separated from each other by intact membranes. (B) Several distended astrocytic processes (asterisks) of the perivascular glial limitans are present around a blood vessel Lu: lumen. (C) On higher magnification of boxed area from (B), the endothelial basement is shown to be filled with irregularly orientated amyloid fibrils (arrows). (D) The earliest stage of vascular amyloid is shown. Here the endothelial basement membrane (black arrowheads) is intact, but the pericyte basement membrane (black arrows) is thickened and heavily infiltrated with short amyloid fibrils (white arrowheads). (E) Severe neuritic dystrophy in which several processes show an excessive accumulation of organelles and abnormal electron dense bodies. (F) White matter of the cerebellum showing an empty myelin sheath (seen as vacuoles by light microscopy) and also a dark degenerate axon (asterisk) within an intact myelin sheath. Similar findings were observed in RML scrapie-infected tg44+/+ mice. Scale bars: A, 2 µm; B, E, F, 1 µm; C, 500 nm; D, 500 nm.

Using staining with uranyl acetate/lead citrate large areas of distended swollen processes could be seen (Figure 6A), which were similar to the areas of immunogold-labeled PrPres shown above (Figure 5G). At higher magnification swollen perivascular glial processes were often seen (Figure 6B), and fibrils were visible in the endothelial basement membrane (Figure 6C) and/or pericyte basement membrane (Figure 6D). The initial site of aggregation into fibrils was in the ablumenal basement membranes (Figure 5D). Dystrophic neurites were also frequently noted in gray matter (Figure 6E). These were most conspicuous surrounding perivascular amyloid plaques and corresponded to sites of APP labeling. In white matter we observed degeneration of axons, including empty distended myelin sheaths (Figure 6F) (Table 3) which could be seen as white matter vacuoles by light microscopy (Figure 4F).

In contrast to transgenic mice, infected C57BL/10 mice at the time of clinical disease had numerous TSE vacuoles with broken or “hanging” membranes (not shown) [23] [24]. Such vacuoles were never seen in infected transgenic mice (Table 3). In C57BL/10 mice other ultrastructural hallmarks specific for classical prion diseases including membrane accumulation of disease-specific PrPres and TSE-specific membrane alterations were also seen, as reported previously in other prion disease models [17] [7],[18],[24] (Table 3). However, none of these prion disease-specific features was seen in infected tg44+/+ mice. The ultrastructural differences between scrapie-infected C57BL/10 and tg44+/+ mice supported the conclusion that the pathogenesis of disease in these transgenic mice was not typical TSE/prion disease.

### Brain graft experiments

The reasons for the different types of scrapie-induced pathogenesis in C57BL/10 mice and anchorless PrP transgenic mice are not known. Two possibilities include: first, PrPres amyloid and diffuse non-amyloid PrPres might have different neurotoxic effects; second, PrPsen anchoring might influence neurotoxicity induced by infection.

**Figure 7:**
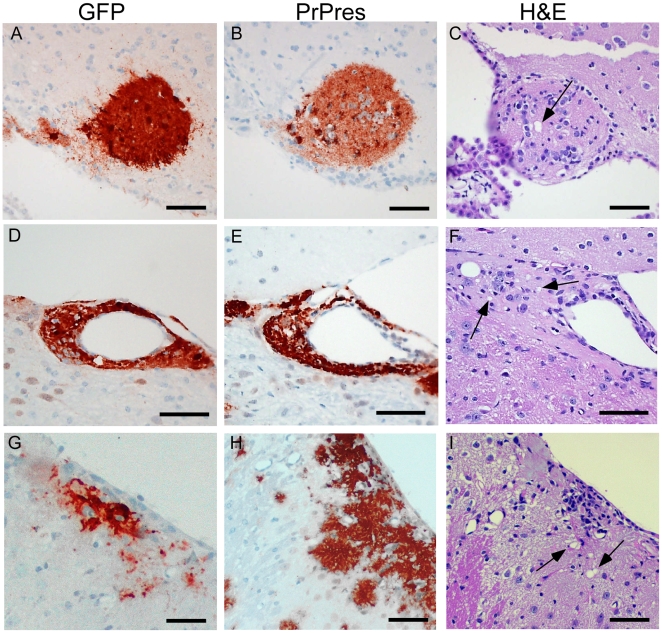
Detection of PrPres and vacuolation in brain tissue of PrPnull and tg44+/− mice with C57BL/6 brain grafts. Grafts expressed green fluorescent protein (GFP), and mice were infected IC with 22L scrapie approximately 5 weeks after grafting. Panels A–C, PrPnull recipient at 261 dpi; Panels D–F, tg44+/− recipient at 261 dpi; Panels G–I, tg44+/− recipient at 200 dpi. Panels A, D, G show staining with anti- GFP which detects constitutive GFP expression in the C57BL/6 donor tissue. Panels B, E, H show D13 staining of PrPres. Panels C, F, I show H&E staining to detect scrapie-induced vacuoles indicated by arrows.

To test whether PrPres derived from GPI-anchored PrPsen could induce gray matter vacuoles in tissue expressing anchorless PrPsen, brain tissue from C57BL/6 mice at embryonic day E12–E14, which expressed green fluorescent protein constitutively in all tissues [25], was grafted into the brain of adult tg44+/− mice or PrP null mice as controls [26]. One month after grafting, mice were infected IC with scrapie, and at 132–511 dpi the brain tissue was examined by histopathology. Recipients had from 1–6 detectable grafts per mouse (Table 4). Representative grafts are shown in Figure 7.

**Table 4:** Studies of PrPres and gray matter vacuolation in scrapie-infected anchorless PrP tg44+/− mice with C57BL/6 embryonic brain grafts.

	Recipient host genotype	
	PrPnull	Tg44+/−
Number of mice	7	10
Number of grafts	20	31
Grafts with PrPres	20	25
Grafts with gray matter vacuoles	18	22
Adjacent host gray matter with PrPres	0	20
Adjacent host gray matter with vacuoles	0	0

Anchorless PrP tg44+/− mice or PrPnull (−/−) adult mice were grafted with 12–14 d embryonic brain tissue from C57BL/6 mice expressing normal anchored PrP and green fluorescent protein (GFP) [25], as described in the Methods. One month after grafting, mice were infected with scrapie by IC inoculation. Mice were euthanized at various times from 135–511 dpi, and brain tissue sections were examined by H&E staining and immunohistochemical staining for GFP and PrPres.

At 261 dpi in the control PrPnull recipient, C57BL/6 graft tissue, identified by presence of green fluorescent protein (GFP) (Figure 7A), had easily detectable PrPres (Figure 7B) present both within the graft and at the interface between the graft and the host tissue, but PrPres did not appear to spread extensively into the PrPnull tissue. TSE gray matter vacuolation was seen only within the graft tissue (Figure 7B and 7C). This was similar to a previous report [26].

In tg44+/− recipient mice receiving C57BL/6 grafts, PrPres and gray matter vacuolation was also seen in the graft (Figure 7F and 7I). In the adjacent host tissue expressing anchorless PrP, amyloid PrPres and white matter vacuoles were noted; however, the C57BL/6 PrPres present at the edges of the graft appeared to be unable to induce gray matter vacuoles in the adjacent transgenic tissue (Figure 7F). In some cases the graft cells were not well-demarcated from the host (Figure 7G), and it was not clear whether the PrPres and vacuoles were in the graft or the host (Figure 7H and 7I). These results were representative of observations in 25 grafts in tg44+/− recipients where PrPres was detected in the graft (Table 4). In summary, we found no grafts where expression of anchored PrPres from the C57BL/6 graft could be associated with gray matter spongiosis in adjacent transgenic host tissue. This result suggested that expression of anchored PrPsen in gray matter might be a fundamental requirement for the induction of the typical TSE/prion disease pathogenic process.

## Discussion

In the present experiments scrapie infection of transgenic mice expressing anchorless PrP resulted in a slow fatal brain disease. These results demonstrated new mechanisms of prion-induced pathogenesis associated with the presence of PrPres amyloid and the absence of GPI-anchored PrP. This disease lacked gray matter spongiosis and differed in this respect from scrapie infection in non-transgenic mice, where the disease is characterized by extensive gray matter spongiosis and non-amyloid PrPres deposition.

The current results raised the question of how lack of GPI-linked membrane anchoring of PrP might facilitate formation of PrPres amyloid. GPI anchorless PrP has a longer biological half-life [27] and is secreted by the cell. Both of these attributes might allow more effective and extensive interactions between soluble PrP molecules. In addition, the minimal amount of carbohydrates and the absence of the GPI group on anchorless PrP might favor amyloidogenic hydrophobic protein-protein interactions, particularly at a time of partial protein unfolding during PrP conversion. These features of anchorless PrP are likely to contribute to its enhanced tendency to form amyloid during conversion to PrPres. Anchorless protease-resistant PrP, cleaved at residue 228, comprises 15% of the PrPres in hamster scrapie brain extracts [28], but it is unclear whether this material contributes to the amyloid PrP seen in this model.

Our results differed from those of two interesting mouse prion disease models where PrPres was also found almost entirely in an amyloid form. In the GSS PrP-8kd model [29] and the G3-ME7 model [30], which both used PrP mutant mice, PrP amyloid was seen primarily in the corpus callosum, but did not spread significantly to other brain regions. There was no clinical disease in these models, and transmission experiments suggested very low infectivity titers in the GSS PrP-8kd model. Compared to these two models, the three main distinguishing features of the anchorless PrP model are the ability of the PrPres amyloid to accumulate widely throughout the brain (Figure 4), the resulting fatal brain disease (Figure 2), and the high titer of transmissible agent (Table 2) [31].

The association of amyloid deposition without gray matter spongiosis in our system is reminiscent of the neuropathology seen in certain human familial prion diseases. For example, GSS patients with PrP mutations Y145Stop and Y163Stop had both CAA and parenchymal perivascular amyloid without gray matter spongiosis [32] [33]. Both these mutations result in C-terminally truncated PrP lacking the GPI anchor. Parenchymal amyloid deposition without gray matter spongiosis has also been seen in GSS patients with several other PrP mutations including P102L, P105L, A117V and F198S [6]. Recently two human GSS patients with new PrP mutations producing nonsense codons at positions 226 and 227 were described [34]. Both patients had widespread PrPres amyloid deposition in the absence of gray matter spongiosis, and one had CAA. These patients expressed a nearly full-length form of PrP lacking 6–7 C-terminal residues and the GPI anchor, which was quite similar to the PrP expressed in our anchorless PrP tg mice.

In many GSS patients, amyloid PrPres purified from brain was truncated resulting in a 7–11 kDa protease-resistant fragment from the central region of PrP (approximately residues 81–150)[6],[22],[34]. Interestingly, presence of this truncation has been correlated with the lack of gray matter spongiosis [8],[9]. In contrast, based on previous immunoblot studies, the proteinase K-resistant PrPres amyloid in our model appeared to contain residues 88–231 [19], which was similar to the PrPres found in human and animal prion diseases with extensive gray matter spongiosis. Furthermore, PrPres in tissue sections could be stained with anti-PrP serum R24, specific to residues 23–37 (data not shown) suggesting that there was no significant truncation at the N-terminus beyond the signal peptide. Thus, lack of spongiosis in our model appeared dependent on the absence of GPI-anchoring rather than truncation of the PrPres.

Two possibilities might explain the correlation between lack of GPI- anchored PrP and lack of gray matter spongiosis in our infected transgenic mice: (1) anchorless amyloid PrPres might be less neurotoxic than diffuse PrPres, and/or (2) anchored PrPsen might be required for PrPres-mediated neurotoxic membrane interactions. The former explanation could not be proven or excluded by our results. However, the latter interpretation was supported by data from brain graft experiments. After scrapie infection of tg44+/− mice grafted with C57BL/6 brain expressing normal anchored PrPsen, we observed gray matter spongiosis and non-amyloid PrPres deposition in C57BL/6 grafts, but not in adjacent host tissue expressing only anchorless PrPsen. Tissue expressing only anchorless PrPsen appeared to be unable to respond to the presence of GPI-anchored PrPres produced in the nearby grafts, and no gray matter spongiosis was produced. Therefore, lack of anchored PrPsen might by itself explain the lack of gray matter spongiosis in transgenic mice.

However, even in the absence of anchored PrPsen, the amyloid PrPres was able to induce additional pathogenic processes capable of causing fatal neurological disease. By both light and electron microscopy we observed evidence for three distinct pathogenic processes not seen in typical prion disease in C57BL/10 mice (Box 1):

(1) Brain damage caused by tissue distortion by large amyloid plaques. These plaques were associated with neuronal loss, axonal pathology and gliosis (Figure 4E, H–L Figure 6A,E,F). The more rapid accumulation of PrPres in tg44+/+ mice compared to tg44+/− mice (Figure 3) suggested a faster growth of large space-occupying plaques which might explain in part the clinical neurological signs leading to death of tg44+/+ and tg23+/+ mice (Figure 2).

(2) A second pathogenic process in scrapie-infected transgenic mice was suggested by ultrastructural studies finding that the early aggregation of PrPres into fibrillar amyloid was located at or within vascular basement membranes (Figures 5C, 5D, 6C, 6D). This was associated with vascular damage including occlusion (Figure 5B), amyloid replacement of basement membrane and tunica media, and occasional micro-hemorrhages. This pathology was similar to that observed in CAA seen in Alzheimer's disease and several familial amyloid diseases including two prion diseases [14],[15],[32],[33].

(3) Evidence of a third pathogenic process in the transgenic mice was suggested by finding of small deposits of immunogold-labeled PrPres at the ultrastructural level in the extracellular spaces between glial and neuritic processes in gray matter (Figures 5D, 5E, 5F). These PrPres deposits were small, and there was no distortion of the extracellular space or visible aggregation into amyloid fibrils. However, the adjacent processes were often highly dystrophic (Figure 6E) or swollen and devoid of organelles, and they appeared to coalesce to form empty spaces larger than the original processes (Figures 5G, 6A, 6B). These abnormal areas, which were also noted in heterozygous tg44+/− and tg23+/− mice, appeared to represent a form of damage related to small, rather than large, PrPres deposits, and they did not require the presence of anchored PrPsen for their formation.

**Box 1. Three neuropathogenic processes found in scrapie-infected homozygous anchorless PrP transgenic mice (tg44+/+ and tg23+/+)**

1. Displacement of brain structure by rapidly expanding amyloid plaques
  - Associated with neuronal dropout and adjacent axonal and neurite damage
2. Early PrPres amyloid on or within basement membranes of endothelial cells, smooth muscle cells and pericytes
  - Possible damage to basement membrane and obstruction of the flow of interstitial brain fluid by PrPres amyloid
  - Possible role of basement membrane components in assisting PrPres formation, e.g. glucosaminoglycans, collagen, laminin, etc.
  - Vascular occlusion and occasional micro-hemorrhages
3. Accumulation of PrPres in the interstitial space between neurite and glial processes
  - Neuronal and glial toxicity with formation of swollen neurites and glial processes with fewer organelles

The early localization of PrPres at basement membranes (Figures 5A, C, D), suggested that the PrP conversion process might initiate at these sites, and implied that basement membrane molecules might facilitate PrP conversion. For example, basement membrane might filter or trap soluble PrPsen molecules or small PrPres oligomers from the extracellular interstitial fluid of brain increasing their local concentration, thus favoring conversion to larger PrPres amyloid aggregates. Serum amyloid P-component which binds to all amyloids and is a constituent of basement membranes might also contribute to local PrP conversion [35]. In addition, collagen, laminin and heparin sulfate-containing proteoglycans are major components of basement membranes, and PrP can bind to both the laminin receptor and heparan sulfate which can associate directly or indirectly with PrP [36]–[38]. Heparan sulfate and other glycosaminoglycan (GAG) moieties can delay scrapie disease in vivo [39]–[42] [43],[44], and some GAG molecules can alter PrP conversion in vitro [45]. A scaffolding mechanism might account for this effect. For example, soluble anchorless PrPsen monomers might be held in place by GAG polymers to increase local concentration and facilitate conversion by PrPres, analogous to the tethering of anchored PrPsen on cell membranes [46],[47]. In addition, attachment of small mobile PrPres oligomers to GAG polymers might assist conversion at the basement membrane. Subsequently newly formed larger less mobile PrPres could serve as an efficient scaffold for further conversion allowing the process to extend out into the brain parenchyma. Eventually this process might form very large PrPres amyloid plaques with blood vessels at the center as we observed (Figure 4E and 5A).

The vascular amyloid pathology seen in our scrapie-infected transgenic mice (Figures 4E, 5A–D, 6B–D) was similar to CAA seen in Alzheimer's disease as well as several familial amyloid diseases [14], including two forms of familial prion disease [32] [14]. In Alzheimer's disease, amyloid fibrils within vascular basement membranes are thought to impede interstitial fluid drainage leading to an increase in Aβ concentrations within the extracellular space. Such increased soluble Aβ and oligomeric proto-amyloid fragments are considered a likely contributory factor in the cognitive decline of Alzheimer's disease patients [15]. Similar processes might contribute to the clinical disease seen in the anchorless PrP scrapie model. Since all these diseases with CAA show amyloid localization with basement membranes, drugs capable of blocking amyloid-basement membrane interactions might be effective treatments for some of these diseases. In the case of prion diseases, one such compound, pentosan polysulfate, a small GAG oligomer, was effective in blocking PrPres generation in an infected cell line [48] and delayed onset of clinical scrapie in vivo [41] [42] [44]. Similarly a decoy molecule preventing PrP interaction with the laminin receptor (LRP/LR) reduced PrPres levels and delayed disease in vivo [37]. Determining the precise glycans and proteins involved in the protein interactions leading to amyloid deposition in all the CAA diseases might be important in designing new therapeutic approaches.

## Methods

### Mice

Ethics statement: All mice were housed at the Rocky Mountain Laboratories (RML) in an AAALAC-accredited facility, and research protocols and experimentation were approved by the NIH RML Animal Care and Use Committee.

C57BL/10SnJ mice (Prnp+/+) were obtained from Jackson Laboratories (Bar Harbor, Maine). C57BL/10SnJ PrP−/− mice were created at RML by crossing 129/Ola PrP−/− mice [49] with C57BL/10SnJ mice, followed by nine serial backcrosses to C57BL/10SnJ with selection for the Prnp+/− genotype using previously described PCR reactions to detect both the Prnp+ and Prnp null alleles [19]. One intercross was then done, and C57BL/10SnJ Prnp−/− (PrP−/−) mice were selected and interbred. Heterozygous Prnp+/− mice were obtained by intercrossing C57BL/10 (Prnp+/+) mice with C57BL/10 Prnp−/− mice.

Transgenic GPI anchorless PrP mice (tg44+/− and tg23+/−) were made as described previously [19] and then backcrossed to C57BL/10SnJ-Prnp−/− mice for six to nine generations with selection for the Prnp−/− genotype and the tg44 or 23+/− genotype. Thus these mice contained one anchorless PrP transgene allele and did not express any normal anchored mouse PrP allele. Heterozygous transgene lines tg23+/− and tg44+/− were each interbred to create homozygous lines (tg44+/+ and tg23+/+). Offspring were tested for transgene zygosity using real-time DNA PCR on an ABI Prism 7900 HT Sequence detection system and SDS 2.2.2 software. The following probes and primers were designed to amplify the mouse Prnp sequence: probe (moPrPlower418T): (5′-CGGTCCTCCCAGTCGTTGCCAAA), forward primer (moPrP-396F): (5′-CGTGAGCAGGCCCATGATC), reverse primer (moPrP-465R): (5′GCGGTACATGTTTTCACGGTAGT). Individual mice identified by rtPCR as transgene homozygous were then bred to Prnp−/− mice to confirm homozygosity. Homozygous mice were then interbred to create additional mice for experimentation. Both tg44 and tg23 lines were used in the present experiments to demonstrate that the observed findings were consistent with transgene expression rather than a result of an integration site artifact.

### Scrapie infections

Four to six week old mice were inoculated intracerebrally with 50 µl of a 1% brain homogenate of 22L or RML scrapie containing 0.7–1.0×10^6^ ID_50_. One ID_50_ is the dose causing infection in 50% of C57BL/10 mice. Animals were observed daily for onset and progression of scrapie. Mice were euthanized when clinical signs were consistent and progressive. Signs differed somewhat in C57BL/10 and tg44+/+ and tg23+/+ mice (Table 1). In heterozygous tg44+/− and tg23+/− mice many mice developed signs of debilitation such as weight loss, dermatitis and infections requiring euthanasia prior to severe neurological signs.

### Immunoblotting

For detection of PrPsen from uninfected brains, tissues were homogenized (20% w/v) using a bead beater in ice-cold 0.01 M Tris-HCl pH 7.6 containing protease inhibitors (10 µM leupeptin, 1 µM pepstatin, and 1 µM aprotinin). Each sample was vortexed for 1 minute followed by sonication for 1 minute. Insoluble debris was removed by centrifugation at 2700 g for 10 minutes at 4°C. Samples were mixed 1∶1 with 2X SDS-PAGE sample buffer and boiled for 3–5 minutes. PNGase F reactions were done using 4.4 mg tissue equivalents in a total volume of 20 µl SDS-PAGE sample buffer [50]. Samples were serially diluted two-fold in sample buffer to give the amount of brain tissue (mg brain equivalents) indicated for each lane. Immunoblots were probed by using monoclonal anti-PrP D13 at a dilution of 1∶5000 (InPro Biotechnology, South San Francisco, CA), followed by secondary antibody sheep anti-human Ig (dilution 1∶5000) (GE Healthcare, formerly Amersham Biosciences, Piscataway, NJ) and enhanced chemiluminescence according to the manufacturers instructions (Amersham-Pharmacia, Uppsala, Sweden).

For detection of PrPres either with or without PNGase F, samples were prepared as described [51]. Blots were probed as described above.

### Brain grafting

Embryonic brain tissue was obtained from E12–E14 C57BL/6 embryos which expressed green fluorescent protein (GFP) in all tissues [25]. Mice were purchased originally from Jackson laboratories and were bred at Rocky Mountain Laboratories by Dr. Kim Hasenkrug. Pregnant mothers were euthanized and embryos dissected with forceps in media under a dissecting microscope to obtain the mesencephalon and telencephalon. Tissue was partially disrupted by pipetting to generate small fragments. This suspension (30 µl) was inoculated intracerebrally through the skull into the parietal brain region of 3–4 week old PrPnull mice or tg44+/− mice. One month later recipient mice were infected intracerebrally with 22L scrapie as described above. At various times thereafter mice were euthanized and brain tissue was examined histologically for GFP and PrPres by specific immunohistochemistry and for typical scrapie-induced gray matter spongiosis by H&E staining.

### Histopathology and immunohistochemistry

Mice were euthanized and brains were placed in 3.7% phosphate-buffered formalin for 3 to 5 days before dehydration and embedding in paraffin. Serial 4 µm sections were cut using a standard Leica microtome, placed on positively charged glass slides and dried overnight at 56°C. Slides were stained with a standard protocol of hematoxylin and eosin (H&E) for observation of overall pathology. For PrPres detection, slides were rehydrated in 0.1 M citrate buffer, pH 6.0 and then heated at 120°C, 20 psi for 20 minutes in a decloaking chamber (Biocare, Walnut Creek, CA). Immunohistochemical staining was performed using the Ventana automated Nexus stainer (Ventana, Tucson, AZ). Staining for PrP used a standard avidin-biotin complex immunoperoxidase protocol using anti-PrP antibody D13 (In-Pro Biotechnology, South San Francisco, CA) at a dilution of 1∶500 and incubated at 4°C for 16 hours. Biotinylated goat anti-human IgG (Jackson Immuno Research, West Grove, PA) was used at a 1∶500 dilution as the secondary antibody. Detection was performed with Ventana streptavidin-alkaline phosphatase with Fast Red chromogen. Tissue sections for microglia staining were pretreated and stained with anti-Iba1 as described [52] except that detection was done using the Ventana Fast Red chromagen as above. Astroglia were stained with anti-GFAP as described [52], and detection was completed with Ventana streptavidin-alkaline phosphatase using Fast Red. Tissue sections for staining with anti-amyloid precursor protein (APP) were pretreated as described for anti-PrP antibody D13. Anti-APP (Zymed Laboratories, San Francisco, CA) was used at a 1∶500 dilution followed by a 1∶250 dilution of biotinylated-goat anti-rabbit IgG (Vector Laboratories, Burlington, CA), and detection with Ventana streptavidin-horseradish peroxidase plus amino ethyl carbazol (AEC) chromagen. Staining of phosphorylated neurofilament proteins was performed using a monoclonal antibody cocktail pan-axonal neurofilament marker SMI-312 (Covance, Princeton, NJ) at a 1∶250 dilution. Monoclonal antibody to nonphosphorylated neurofilament proteins was also used (SMI-311). Primary antibodies were followed by biotinylated horse anti-mouse IgG secondary antibody at a 1∶250 dilution. Ventana AEC reagent was used for detection. Green fluorescent protein (GFP) was detected using a mixture of two mouse anti-GFP monoclonal antibodies (clones 7.1 and 13.1) at dilution of 1∶200 (Roche Applied Science, Indianapolis, IN), followed by biotinylated horse anti-mouse IgG (Vector Laboratories, Burlington, CA) at a dilution of 1∶250 and detected with AEC chromogen (Ventana) as described above. All histopathology slides were read using an Olympus BX51 microscope and images were obtained using Microsuite FIVE software.

### Perfusion/Processing for electron microscopy

Mice were perfused with fixative containing 3% paraformaldehyde and 1% glutaraldehyde in PBS. Excised tissues were then immersed in this fixative and held overnight at 4 degrees C. Tissue pieces were processed further using a Lynx® automated tissue processor with agitation as follows: one wash in PBS for 3 hr at 20 degrees, one wash in 0.1 M sodium phosphate buffer pH 7.2 at 20 degrees for 4 hr, post-fix in 2% osmium tetroxide in phosphate buffer at 20 degrees for 6 hr, one wash in phosphate buffer at 20 degrees for 3 hr, three washes in water at 20 degrees for 3 hr each, in-block staining with 1% uranyl acetate in water at 20 degrees for 6 hr, 3 washes in water at 20 degrees for 3 hr each, dehydration in 70%, 100%, and 100% acetone at 10 degrees for 3 hr each, and infiltration at 20 degrees in Araldite resin (Structure Probe, Inc., West Chester, PA) at 50% for 8 hr, 75% for 12 hr, and two changes of 100% for 20 hr each. Further tissue blocks were processed using a Leica EM TP processor using the procedure above with the omission of the uranyl acetate. Tissue blocks were then transferred to fresh resin in molds and polymerized at 65 degrees for 24 to 48 hr.

### Immunolabelling of resin block sections for light microscopy

Thick (1 µm) sections were stained by toluidine blue or were immunolabelled using the avidin-biotin technique. Sections were deplasticized with saturated sodium ethoxide for up to 30 minutes. Endogenous peroxidase was blocked and sections were de-osmicated with 6% hydrogen peroxide for 10 minutes, followed by pre-treatment with neat formic acid for 5 minutes. Normal serum was then applied for 1 hour to block non-specific labeling. 1A8 anti-PrP serum [53] at a dilution of 1∶6000, or pre-immune serum were then applied for 15 hours and reaction product developed using 3-3′ diaminobenzidine.

### Immunolabeling for electron microscopy

For routine electron microscopy areas were selected from 1 µm thick toluidine blue stained sections and counterstained with uranyl acetate and lead citrate. For ultrastructural immunohistochemistry, serial 65 nm sections were taken from blocks previously identified from immuno-labeled 1 µm thick sections as described above. The 65 nm sections were placed on 600 mesh gold grids and etched in sodium periodate for 60 minutes. Endogenous peroxidase was blocked and sections de-osmicated with 6% hydrogen peroxide in water for 10 minutes followed by enhancement of antigen expression with formic acid for 10 minutes. Residual aldehyde groups were quenched with 0.2 M glycine in PBS, pH 7.4 for 3 minutes. Preimmune serum or anti-PrP primary antibody 1A8 [53] or R30 [54] at a 1∶500 or 1∶1500 dilution respectively in incubation buffer were then applied for 15 hours. After rinsing extensively, sections were incubated with Auroprobe 1 nm colloidal gold diluted 1∶50 in incubation buffer for 2 hours. Sections were then post-fixed with 2.5% glutaraldehyde in PBS and labeling enhanced with Goldenhance (Universal Biologicals, Cambridge, UK) for 10 minutes. Grids were counterstained with uranyl acetate and lead citrate.

## References

[1] Aguzzi A, Polymenidou M (2004). Mammalian prion biology: one century of evolving concepts.. Cell.

[2] Di Bari MA, Chianini F, Vaccari G, Esposito E, Conte M (2008). The bank vole (Myodes glareolus) as a sensitive bioassay for sheep scrapie.. J Gen Virol.

[3] Nonno R, Di Bari MA, Cardone F, Vaccari G, Fazzi P (2006). Efficient transmission and characterization of Creutzfeldt-Jakob disease strains in bank voles.. PLoS Pathog.

[4] Jeffrey M, Goodsir CM, Bruce ME, McBride PA, Fraser JR (1997). In vivo toxicity of prion protein in murine scrapie: ultrastructural and immunogold studies.. Neuropathol Appl Neurobiol.

[5] Gonzalez L, Martin S, Begara-McGorum I, Hunter N, Houston F (2002). Effects of agent strain and host genotype on PrP accumulation in the brain of sheep naturally and experimentally affected with scrapie.. J Comp Pathol.

[6] Ghetti B, Piccardo P, Frangione B, Bugiani O, Giaccone G (1996). Prion protein amyloidosis.. Brain Pathol.

[7] Ersdal C, Goodsir CM, Simmons MM, McGovern G, Jeffrey M (2009). Abnormal prion protein is associated with changes of plasma membranes and endocytosis in bovine spongiform encephalopathy (BSE)-affected cattle brains.. Neuropathol Appl Neurobiol.

[8] Parchi P, Chen SG, Brown P, Zou W, Capellari S (1998). Different patterns of truncated prion protein fragments correlate with distinct phenotypes in P102L Gerstmann-Straussler-Scheinker disease.. Proc Natl Acad Sci U S A.

[9] Piccardo P, Dlouhy SR, Lievens PM, Young K, Bird TD (1998). Phenotypic variability of Gerstmann-Straussler-Scheinker disease is associated with prion protein heterogeneity.. J Neuropathol Exp Neurol.

[10] Piccardo P, Seiler C, Dlouhy SR, Young K, Farlow MR (1996). Proteinase-K-resistant prion protein isoforms in Gerstmann-Straussler-Scheinker disease (Indiana kindred).. J Neuropathol Exp Neurol.

[11] Giaccone G, Verga L, Bugiani O, Frangione B, Serban D (1992). Prion protein preamyloid and amyloid deposits in Gerstmann- Straussler- Scheinker disease, Indiana kindred [published erratum appears in Proc Natl Acad Sci U S A 1993 Jan 1;90(1):302].. Proc Natl Acad Sci U S A.

[12] Bruce ME, Dickinson AG (1985). Genetic control of amyloid plaque production and incubation period in scrapie-infected mice.. J Neuropathol Exp Neurol.

[13] Jeffrey M, Goodsir CM, Bruce ME, McBride PA, Scott JR (1992). Infection specific prion protein (PrP) accumulates on neuronal plasmalemma in scrapie infected mice.. Neurosci Lett.

[14] Revesz T, Ghiso J, Lashley T, Plant G, Rostagno A (2003). Cerebral amyloid angiopathies: a pathologic, biochemical, and genetic view.. J Neuropathol Exp Neurol.

[15] Weller RO, Subash M, Preston SD, Mazanti I, Carare RO (2008). Perivascular drainage of amyloid-beta peptides from the brain and its failure in cerebral amyloid angiopathy and Alzheimer's disease.. Brain Pathol.

[16] Stahl N, Borchelt DR, Prusiner SB (1990). Differential release of cellular and scrapie prion proteins from cellular membranes by phosphatidylinositol-specific phospholipase C.. Biochemistry.

[17] Jeffrey M, Goodsir CM, Bruce M, McBride PA, Scott JR (1994). Correlative light and electron microscopy studies of PrP localisation in 87V scrapie.. Brain Res.

[18] Jeffrey M, McGovern G, Goodsir CM, Siso S, Gonzalez L (2009). Strain-associated variations in abnormal PrP trafficking of sheep scrapie.. Brain Pathol.

[19] Chesebro B, Trifilo M, Race R, Meade-White K, Teng C (2005). Anchorless prion protein results in infectious amyloid disease without clinical scrapie.. Science.

[20] Prusiner SB, Scott M, Foster D, Pan KM, Groth D (1990). Transgenetic studies implicate interactions between homologous PrP isoforms in scrapie prion replication.. Cell.

[21] Manson JC, Clarke AR, McBride PA, McConnell I, Hope J (1994). PrP gene dosage determines the timing but not the final intensity or distribution of lesions in scrapie pathology.. Neurodegen.

[22] Tagliavini F, Prelli F, Ghiso J, Bugiani O, Serban D (1991). Amyloid protein of Gerstmann-Straussler-Scheinker disease (Indiana kindred) is an 11 kd fragment of prion protein with an N-terminal glycine at codon 58.. EMBO J.

[23] Jeffrey M, Scott JR, Fraser H (1991). Scrapie inoculation of mice: light and electron microscopy of the superior colliculi.. Acta Neuropathol.

[24] Jeffrey M, Goodsir CM, Race RE, Chesebro B (2004). Scrapie-specific neuronal lesions are independent of neuronal PrP expression.. Ann Neurol.

[25] Okabe M, Ikawa M, Kominami K, Nakanishi T, Nishimune Y (1997). ‘Green mice’ as a source of ubiquitous green cells.. FEBS Lett.

[26] Brandner S, Isenmann S, Raeber A, Fischer M, Sailer A (1996). Normal host prion protein necessary for scrapie-induced neurotoxicity.. Nature.

[27] Campana V, Caputo A, Sarnataro D, Paladino S, Tivodar S (2007). Characterization of the properties and trafficking of an anchorless form of the prion protein.. J Biol Chem.

[28] Stahl N, Baldwin MA, Burlingame AL, Prusiner SB (1990). Identification of glycoinositol phospholipid linked and truncated forms of the scrapie prion protein.. Biochemistry.

[29] Piccardo P, Manson JC, King D, Ghetti B, Barron RM (2007). Accumulation of prion protein in the brain that is not associated with transmissible disease.. Proc Natl Acad Sci U S A.

[30] Tuzi NL, Cancellotti E, Baybutt H, Blackford L, Bradford B (2008). Host PrP glycosylation: a major factor determining the outcome of prion infection.. PLoS Biol.

[31] Race B, Meade-White K, Oldstone MB, Race R, Chesebro B (2008). Detection of prion infectivity in fat tissues of scrapie-infected mice.. PLoS Pathog.

[32] Ghetti B, Piccardo P, Spillantini MG, Ichimiya Y, Porro M (1996). Vascular variant of prion protein cerebral amyloidosis with tau-positive neurofibrillary tangles: the phenotype of the stop codon 145 mutation in PRNP.. Proc Natl Acad Sci U S A.

[33] Revesz T, Holton JL, Lashley T, Plant G, Frangione B (2009). Genetics and molecular pathogenesis of sporadic and hereditary cerebral amyloid angiopathies.. Acta Neuropathol.

[34] Jansen C, Parchi P, Capellari S, Vermeij AJ, Corrado P (2009). Prion protein amyloidosis with divergent phenotype associated with two novel nonsense mutations in PRNP.. Acta Neuropathol.

[35] Dyck RF, Lockwood CM, Kershaw M, McHugh N, Duance VC (1980). Amyloid P-component is a constituent of normal human glomerular basement membrane.. J Exp Med.

[36] Pflanz H, Vana K, Mitteregger G, Pace C, Messow D (2009). Microinjection of lentiviral vectors expressing small interfering RNAs directed against laminin receptor precursor mRNA prolongs the pre-clinical phase in scrapie-infected mice.. J Gen Virol.

[37] Pflanz H, Vana K, Mitteregger G, Renner-Muller I, Pace C (2009). Scrapie-infected transgenic mice expressing a laminin receptor decoy mutant reveal a prolonged incubation time associated with low levels of PrPres.. J Mol Biol.

[38] Caughey B, Brown K, Raymond GJ, Katzenstien GE, Thresher W (1994). Binding of the protease-sensitive form of PrP (prion protein) to sulfated glycosaminoglycan and Congo red.. J Virol.

[39] Kimberlin RH, Walker CA (1986). Suppression of scrapie infection in mice by heteropolyanion 23, dextran sulfate, and some other polyanions.. Antimicrob Agents Chemother.

[40] Ladogana A, Casaccia P, Ingrosso L, Cibati M, Salvatore M (1992). Sulphate polyanions prolong the incubation period of scrapie-infected hamsters.. J Gen Virol.

[41] Diringer H, Ehlers B (1991). Chemoprophylaxis of scrapie in mice.. J Gen Virol.

[42] Doh-ura K, Ishikawa K, Murakami-Kubo I, Sasaki K, Mohri S (2004). Treatment of Transmissible Spongiform Encephalopathy by Intraventricular Drug Infusion in Animal Models.. J Virol.

[43] Farquhar CF, Dickinson AG (1986). Prolongation of scrapie incubation period by an injection of dextran sulphate 500 within the month before or after infection.. J Gen Virol.

[44] Larramendy-Gozalo C, Barret A, Daudigeos E, Mathieu E, Antonangeli L (2007). Comparison of CR36, a new heparan mimetic, and pentosan polysulfate in the treatment of prion diseases.. J Gen Virol.

[45] Wong C, Xiong L-W, Horiuchi M, Raymond LD, Wehrly K (2001). Sulfated glycans and elevated temperature stimulate PrP^Sc^ dependent cell-free formation of protease-resistant prion protein.. EMBO J.

[46] Baron GS, Wehrly K, Dorward DW, Chesebro B, Caughey B (2002). Conversion of raft associated prion protein to the protease-resistant state requires insertion of PrP-res (PrP(Sc)) into contiguous membranes.. EMBO J.

[47] Baron GS, Caughey B (2003). Effect of glycosylphosphatidylinositol anchor-dependent and - independent prion protein association with model raft membranes on conversion to the protease-resistant Isoform.. J Biol Chem.

[48] Caughey B, Raymond GJ (1993). Sulfated polyanion inhibition of scrapie-associated PrP accumulation in cultured cells.. J Virol.

[49] Manson JC, Clarke AR, Hooper ML, Aitchison L, McConnell I (1994). 129/Ola mice carrying a null mutation in PrP that abolishes mRNA production are developmentally normal.. Mol Neurobiol.

[50] Race BL, Meade-White KD, Ward A, Jewell J, Miller MW (2007). Levels of abnormal prion protein in deer and elk with chronic wasting disease.. Emerg Infect Dis.

[51] Meade-White K, Race B, Trifilo M, Bossers A, Favara C (2007). Resistance to chronic wasting disease in transgenic mice expressing a naturally occurring allelic variant of deer prion protein.. J Virol.

[52] Kercher L, Favara C, Striebel JF, LaCasse R, Chesebro B (2007). Prion protein expression differences in microglia and astroglia influence scrapie-induced neurodegeneration in the retina and brain of transgenic mice.. J Virol.

[53] Jeffrey M, Goodsir CM, Fowler N, Hope J, Bruce ME (1996). Ultrastructural Immuno-localization of Synthetic Prion Protein Peptide Antibodies in 87V Murine Scrapie.. Neurodegen.

[54] Caughey B, Raymond GJ, Ernst D, Race RE (1991). N-terminal truncation of the scrapie-associated form of PrP by lysosomal protease(s): implications regarding the site of conversion of PrP to the protease-resistant state.. J Virol.

